# Forecasting seasonal influenza-like illness in South Korea after 2 and 30 weeks using Google Trends and influenza data from Argentina

**DOI:** 10.1371/journal.pone.0233855

**Published:** 2020-07-16

**Authors:** Soo Beom Choi, Insung Ahn

**Affiliations:** 1 Department of Data-centric Problem Solving Research, Korea Institute of Science and Technology Information, Daejeon, Republic of Korea; 2 Center for Convergent Research of Emerging Virus Infection, Korea Research Institute of Chemical Technology, Daejeon, Republic of Korea; Newcastle University, UNITED KINGDOM

## Abstract

We aimed to identify variables for forecasting seasonal and short-term targets for influenza-like illness (ILI) in South Korea, and other input variables through weekly time-series of the variables. We also aimed to suggest prediction models for ILI activity using a seasonal autoregressive integrated moving average, including exogenous variables (SARIMAX) models. We collected ILI, FluNet surveillance data, Google Trends (GT), weather, and air-pollution data from 2010 to 2019, applying cross-correlation analysis to identify the time lag between the two respective time-series. The relationship between ILI in South Korea and the input variables were evaluated with Linear regression models. To validate selected input variables, the autoregressive moving average, including exogenous variables (ARMAX) models were used to forecast seasonal ILI after 2 and 30 weeks with a three-year window for the training set used in the fixed rolling window analysis. Moreover, a final SARIMAX model was constructed. Influenza A virus activity peaks in South Korea were roughly divided between the 51^st^ and the 7^th^ week, while those of influenza B were divided between the 3^rd^ and 14^th^ week. GT showed the highest correlation coefficient with forecasts from a week ahead, and seasonal influenza outbreak patterns in Argentina showed a high correlation with those 30 weeks ahead in South Korea. The prediction models after 2 and 30 weeks using ARMAX models had *R*^*2*^ values of 0.789 and 0.621, respectively, indicating that reference models using only the previous seasonal ILI could be improved. The currently eligible input variables selected by the cross-correlation analysis helped propose short-term and long-term predictions for ILI in Korea. Our findings indicate that influenza surveillance in Argentina can help predict seasonal ILI patterns after 30 weeks in South Korea, and these can help the Korea Centers for Disease Control and Prevention determine vaccine strategies for the next ILI season.

## Introduction

Seasonal influenza forecasts can provide data-driven information that supports influenza prevention and mitigation strategies [1]. Various statistical and machine learning methods have been used to predict influenza patterns using variables related to influenza surveillance, such as internet search query data [2]. The burden of 2013–2014 seasonal influenza in Korea was estimated at 125 million USD, higher than the burden observed in the past [3]. However, there have been few studies on influenza prediction in South Korea; current literature on the subject focuses on short-term forecasts using Google Trends (GT) or data from social network services [4, 5]. Moreover, forecasting seasonal influenza in South Korea has proven difficult, due to the two peaks of influenza activity observed in a season.

Forecast targets for influenza should be chosen with quantitative and meaningful definitions that reflect public health needs [1]. The FluSight series of influenza forecasting challenges in the United States has forecast targets that include both seasonal (onset, peak week, and peak intensity) and short-term targets (forecasts up to 4 weeks), which are selected by the Centers for Disease Control and Prevention in the United States to understand the characteristics of seasonal influenza [6]. However, there are no such official influenza forecasting challenges and systems for forecasting seasonal targets in South Korea. Previously, we forecasted seasonal influenza patterns after 26 weeks in the United States using influenza activity in Australia and Chile, where the seasonal patterns and influenza outbreaks were similar to but preceded those observed in the United States [7].

Time series data have internal structures such as autocorrelation and seasonal variation, which can be used to forecast future values of the data using analyzed patterns in it [8]. The autoregressive moving average (ARMA) and autoregressive integrated moving average (ARIMA) models have been selected to analyze time series data in the clinical domain. Zhang et al. presented the prediction models for seasonal influenza using Google Trends and temperature [9]. They used cross-correlation analysis for the selection of input variables and seasonal ARIMA (SARIMA) models for prediction [9]. However, Zhang’s model could predict relatively short-term lags than our previous model for the United States using surveillance data in other countries. Basile et al. presented ARMA models for short-term prediction of influenza-like illness (ILI) [10], and the ARIMA analysis was employed to forecast malaria incidence in Afghanistan with time-series patterns [11].

In this study, we aimed to identify variables that aid in forecasting seasonal and short-term targets for ILI in South Korea. We were able to achieve this using FluNet surveillance data, Google Trends, and weather and air pollution data. We also successfully suggested prediction models for ILI activity for seasonal targets using SARIMA, including exogenous variables (SARIMAX) models.

## Methods

### Data collection

ILI data were collected from the Korea Centers for Disease Control and Prevention (KCDC) [12]. ILI is defined by the KCDC as the quantitative number of people per week with a fever of 38°C, and a cough or a sore throat per 1,000 outpatients. Influenza surveillance data were collected from the FluNet database of the WHO Global Influenza Surveillance Network [13–15]. These data are uploaded to the FluNet database every week by the countries in the network [13]. The FluNet database contains the following variables reported by 160 countries: influenza transmission zone, number of specimens, number of influenza A and B viruses detected by subtype, and number of influenza-positive viruses [15]. We collected surveillance data from the 160 countries from the 40^th^ week of 2010 until the 52^nd^ week of 2019. The starting point for the study period was 2010, due to a novel pandemic strain (H1N1 pdm09) in 2009 [16]. Missing data were replaced with a zero. Total influenza (INF) was defined as the sum of the number of influenza A and B viruses detected among processed specimens [7].

GT demonstrates the people’s interest in near real-time using Google search engine queries [17]. Further, GT provides information on the volume of searches by country. We included the search keywords *“A hyeong dokgam”* and *“B hyeong dokgam”*, which are Korean words for influenza A virus and influenza B virus, respectively, in South Korea from October 2010 to December 2019, with reference to Woo et al [5]. We defined the total GT as the sum of query data for *“A hyeong dokgam”* and *“B hyeong dokgam”*. We obtained weekly search query data from GT. To reduce noise, the GT values that did not exceed zero for more than two consecutive weeks were replaced with zeros.

Weather data in South Korea were obtained from the National Weather Data Release Portal [18]. We included weekly temperature data and the average values for Seoul in South Korea during each week [19]. The air pollution data for South Korea were obtained from the Seoul Information Communication Plaza [20]. We included weekly air pollution data and the average values for Seoul in South Korea during each week [21].

### Statistical analysis

For the variables included in the two time-series, cross-correlations were analyzed using Pearson’s correlation, with a time lag range of ±30 weeks from the 40^th^ week of 2010 until the 52^nd^ week of 2019, with Bonferroni’s correction. Cross-correlation allows for the time lag between two time-series to be identified [22]. If the higher cross-correlation value was found to have a negative lag, the values of the first series (ILI in South Korea) were correlated with the values of the second series (other variables), and the second series was made to precede the first in lag weeks [7]. The ILI in South Korea was compared to the input variables, and we selected variables with a time lag of -20 weeks or less and a correlation coefficient of 0.6 or more for seasonal forecast targets.

Linear regression analyses (LR) were used to evaluate the relationship between the ILI in South Korea and ILI in selected variables by cross-correlation analysis with a time lag from the 40^th^ week of 2010 to the 52^nd^ week of 2019. LR 1 used the ILI in South Korea after the time lag as the dependent variable and previous seasonal data from South Korea as the independent variable. Two univariate LR 2 models used the ILI in South Korea as the dependent variable; the total GT in South Korea for LR 2 of the forecast after 2 weeks and influenza activity in Argentina for LR2 of the forecast after 30 weeks were designated as the independent variables, respectively. Univariate LR 3 used the ILI in South Korea as the dependent variable and the average vapor pressure for the forecast after 30 weeks as the independent variable. The input variables in LR 4 were selected by cross-correlation analysis.

For time-series modeling, the ILI data were categorized into three terms: the trend, seasonal, and resid attributes [23]. Dickey-Fuller Test and seasonal Mann–Kendall test were performed to verify the stationary and seasonal trend term of the ILI time series, respectively [24]. The periodical terms were investigated using an autocorrelation function (ACF) and partial ACF (PACF) diagrams of ILI data. All statistical analyses were performed using Python 3.6.2 (Python Software Foundation), and *p-*values < 0.05 were considered statistically significant.

### Prediction model

Our prediction models included forecasting the ILI in South Korea after 2 weeks and 30 weeks, separately. ILI forecasts after 2 weeks were defined as hindcasts and nowcasts; the former is the forecast of past conditions due to delays in reporting and data accrual, while the latter is the forecast of the current point [1]. The input variable that functioned as a reference in the ARMAX models after 2 and 30 weeks was the previous seasonal ILI. The input variables of the prediction model for ILI after 2 weeks in South Korea were previous seasonal ILI and total GT. The input variables of a prediction model for ILI after 30 weeks in South Korea were previous seasonal ILI, average vapor pressure, and total INF in Argentina.

The autocorrelation function and partial autocorrelation function were used to determine the autoregressive (AR) and moving average (MA) order. Reference 25 contains a complete description of the ARMA analysis [25]. An ARMA model includes parameters such as *p* of the AR order and *q* of the MA order [26]. After validation of the input variables, we found the final model for forecasting ILI after 30 weeks with the best parameters using the “arma_order_select_ic” from the “statsmodels” package in Python 3.6.2. ARMAX, ARIMAX, and SARIMAX were adopted to select the final model. A SARIMA model includes parameters such as *p* of the AR order, *d* of the differencing, *q* of the MA order, and 52 weeks for seasonality.

### Validation for the prediction model

**Fig 1:**
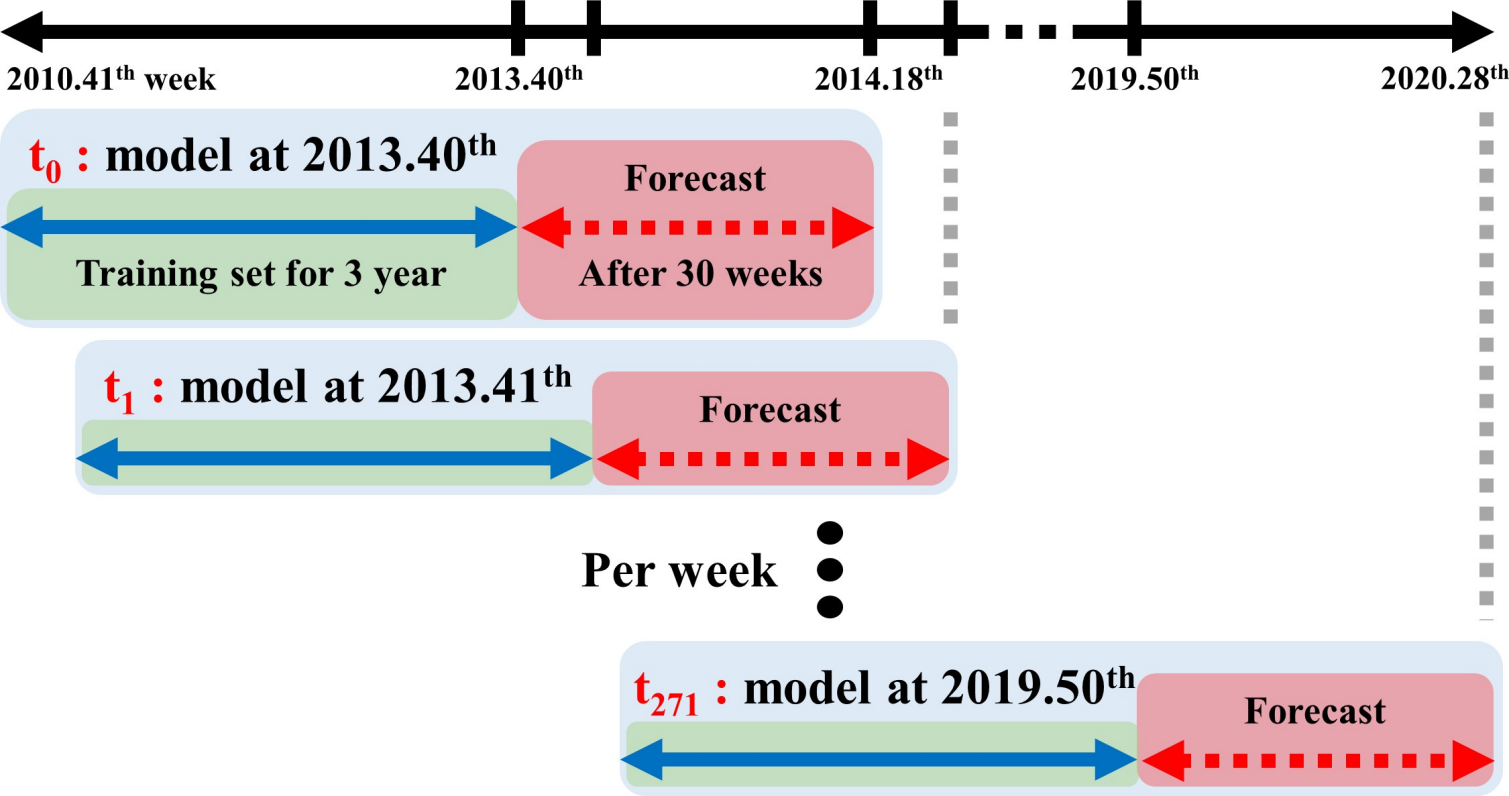
The explanation fixed rolling window analysis with a three-year window for the training set.

For the time-series forecast, we selected a fixed rolling window analysis with a three-year window for the training set and included the forecast values after 2 weeks and 30 weeks for each trial [27]. Five seasons from 2014–2019 were selected to validate the prediction models for seasonal ILI patterns. For example, the forecasted ILI pattern after 30 weeks at the 40^th^ week of 2013 only used the variables from the 40^th^ week of 2010 until the 39^th^ week of 2013 as the training set. The model forecasts ILI at the 18^th^ week of 2014, and this procedure was repeated weekly, as shown in Fig 1.

The coefficient of determination, *R*^2^, which corresponds to the percentage of the variance of the observed time-series that is explained by the model, was calculated. Root-mean-square error (RMSE) was calculated using real and predicted values for ILI activity in the validation set from the 41^st^ week of 2014 to the 52^nd^ week of 2019.

The final model for forecasting ILI was validated using the Akaike Information Criterion (AIC) index. The smaller AIC values corresponded to a better fitting [28]. Ljung–Box test was used to examine the independence of residuals, and Jarque–Bera test was utilized to examine whether the residual of the model followed a normal distribution [29]. Moreover, standardized residual, histogram plus estimated density, normal Q–Q, and ACF of the residual were drawn using the “plot_diagnostics” from the “statsmodels” package in Python 3.6.2. The requirement for ethical approval of this study was waived as we used open-source data that can be downloaded online without login.

## Results

### Cross*-*correlation analysis

**Fig 2:**
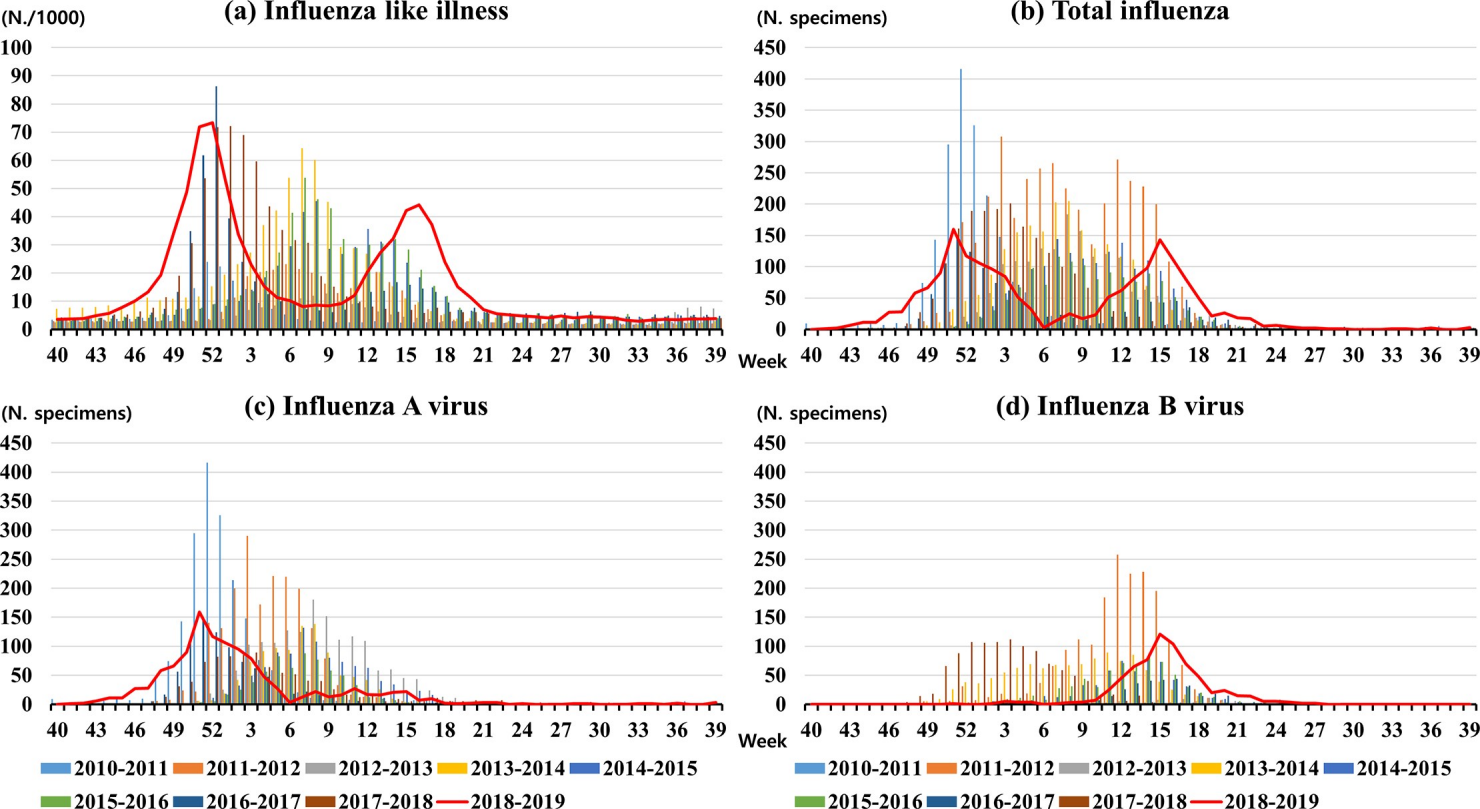
**The nine seasonal patterns of ILI (a), total INF (b), INF A (c), and INF B (d) activity from 2010 to 2019 in South Korea.** The red line denotes influenza cases during the 2018–2019 influenza season, and the bar graphs are for the rest of the seasons. INF, Influenza; ILI, Influenza-like illness.

Fig 2 shows the nine seasonal patterns of ILI, total INF, INF A, and INF B activity from 2010 to 2019 in South Korea. From the 40^th^ week of 2010 to the 39^th^ week of 2019, 17,506 influenza cases from South Korea (INF A, 11,086; INF B, 6,420) were included in this study. The seasonal patterns show irregular characteristics and have one or two peaks of influenza activity. INF A and INF B activity show different patterns. The peak timing of INF A can be roughly divided between the 51^st^ and 7^th^ week, while the peak timing of INF B can be roughly divided between the 3^rd^ and 14^th^ week, as seen in Fig 2.

**Fig 3:**
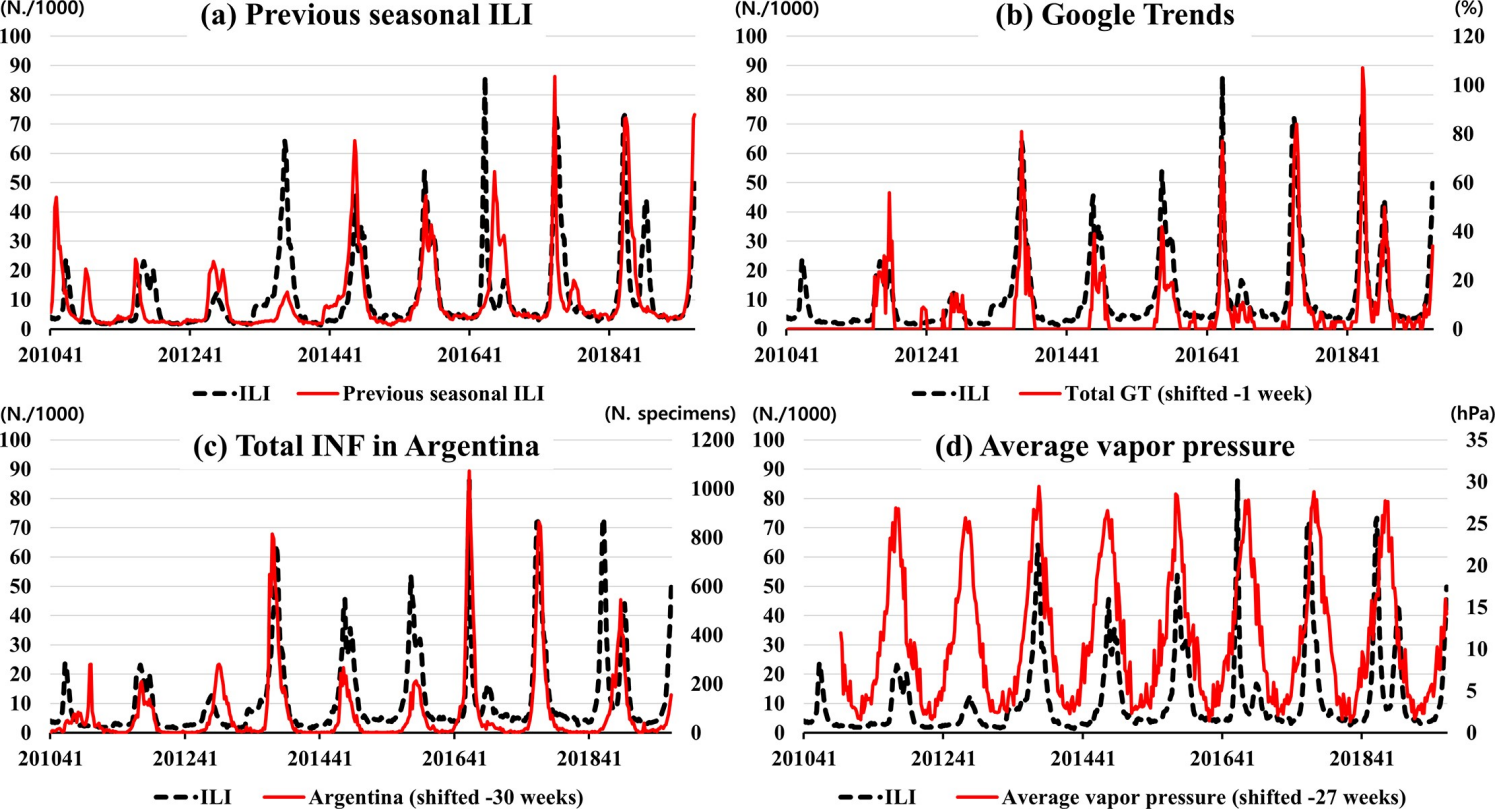
**Correlation between ILI in South Korea and selected variables from 2010 to 2019; previous seasonal ILI (a), Google Trends (b), influenza in Argentina (c), and average vapor pressure (d).** The black line denotes the reference levels, and the red line is input variables. GT, Google Trends; INF, Influenza; ILI, Influenza-like illness.

Table 1 shows the maximum correlation coefficient and the time lag between ILI in South Korea and the input variables using the cross-correlation analysis. The total GT in South Korea had the highest correlation coefficient (0.901) with a -1 week time lag. Total INF activity in Argentina had a high correlation coefficient (0.717) with a -30 week time lag. As a reference, the correlation coefficient for previous seasonal ILI in South Korea was 0.577 with a 0 week time lag. We selected the total GT for forecasting after 2 weeks, and total INF activity in Argentina and average vapor pressure for forecasting after 30 weeks. Fig 3 shows the comparison between ILI in South Korea and selected variables from 2010 to 2019.

**Table 1:** Maximum correlation coefficient and time lag with time series of influenza-like illness and input variables.

Type	Variables	Correlation coefficient	Time lag (Weeks)
Total INF	Argentina*	0.717	-30
	Ireland	0.712	1
	China	0.699	2
	Chile	0.694	25
	Spain	0.692	1
Google Trends	Total GT (INF A + INF B)*	0.901	-1
	Keyword (INF A)	0.825	-1
	Keyword (INF B)	0.691	-1
Weather	Average vapor pressure (hPa)*	0.607	-27
	Average temperature (°C)	0.551	-28
	Relative humidity (%)	0.436	-26
Air-pollution	CO (ppm)	0.426	-4
	PM_10_ (㎍/㎥)	0.374	4
	SO_2_ (ppm)	0.353	-2
Reference	Previous seasonal ILI	0.577	-

GT, Google Trends; INF, Influenza; ILI, Influenza-like illness

* Variables with a correlation coefficient of 0.6 or more and time lag less than -1 weeks

### Linear regression analysis

Table 2 shows the adjusted R-squared values, calculated by LR analyses using ILI in South Korea as dependent variables, and the input variables as independent variables, which were previous seasonal ILI in South Korea, total GT, total INF in Argentina, and average vapor pressure. In LR 1, for the forecasts after 2 weeks and 30 weeks using previous seasonal ILI, the adjusted R-squared was 0.331, which was used as a reference point. The total GT for the forecast after 2 weeks was reported to be 0.744 in LR 2 for forecast after 2 weeks. In LR 2 for the forecast after 30 weeks, the total INF in Argentina shows 0.513, which is higher than the reference point of 0.331. LR 4 reported adjusted R-squared values of 0.794 and 0.712 for the input variables for the forecasts after 2 and 30 weeks, respectively.

**Table 2:** Linear regression analysis for previous seasonal influenza-like illness and selected variables by cross-correlation analysis from the 40^th^ week of 2010 to the 52^nd^ week of 2019.

Forecast week	Variable	LR 1 Beta[P-value]	LR 2 Beta[P-value]	LR 3 Beta[P-value]	LR 4 Beta[P-value]
2 weeks	ILI—South Korea (before 50 week)	0.591[<0.001]			0.255[<0.001]
	Total GT—South Korea (present)		0.789[<0.001]		0.690[<0.001]
	**Adj. R-squared**	**0.331**	**0.744**		**0.794**
30 weeks	ILI—South Korea (before 22 week)	0.591[<0.001]			0.470[<0.001]
	Total INF—Argentina (present)		0.063[<0.001]		0.052[<0.001]
	Average Vapor Pressure—South Korea (present)			0.994[<0.001]	0.047[0.783]
	**Adj. R-squared**	**0.331**	**0.513**	**0.303**	**0.712**

Beta, Beta coefficient; GT, Google Trends; INF, Influenza; ILI, Influenza-like illness; LR, Linear regression

### Prediction models

The parameters (*p*,*q)* of the ARMAX models for prediction after 2 and 30 weeks were selected 1 of *p* and 0 of *q* because we want to validate the input variables without bias of difference in the parameters. Moreover, the fixed rolling window analysis performed 575 models of ARMAX because of two models for the forecast values after 2 and 30 weeks per each day. We selected a simple parameter for ARMAX rather than searching the optimal parameters. The exogenous variables for ARMAX were the selected input variables by cross-correlation and linear regression analyses.

**Fig 4:**
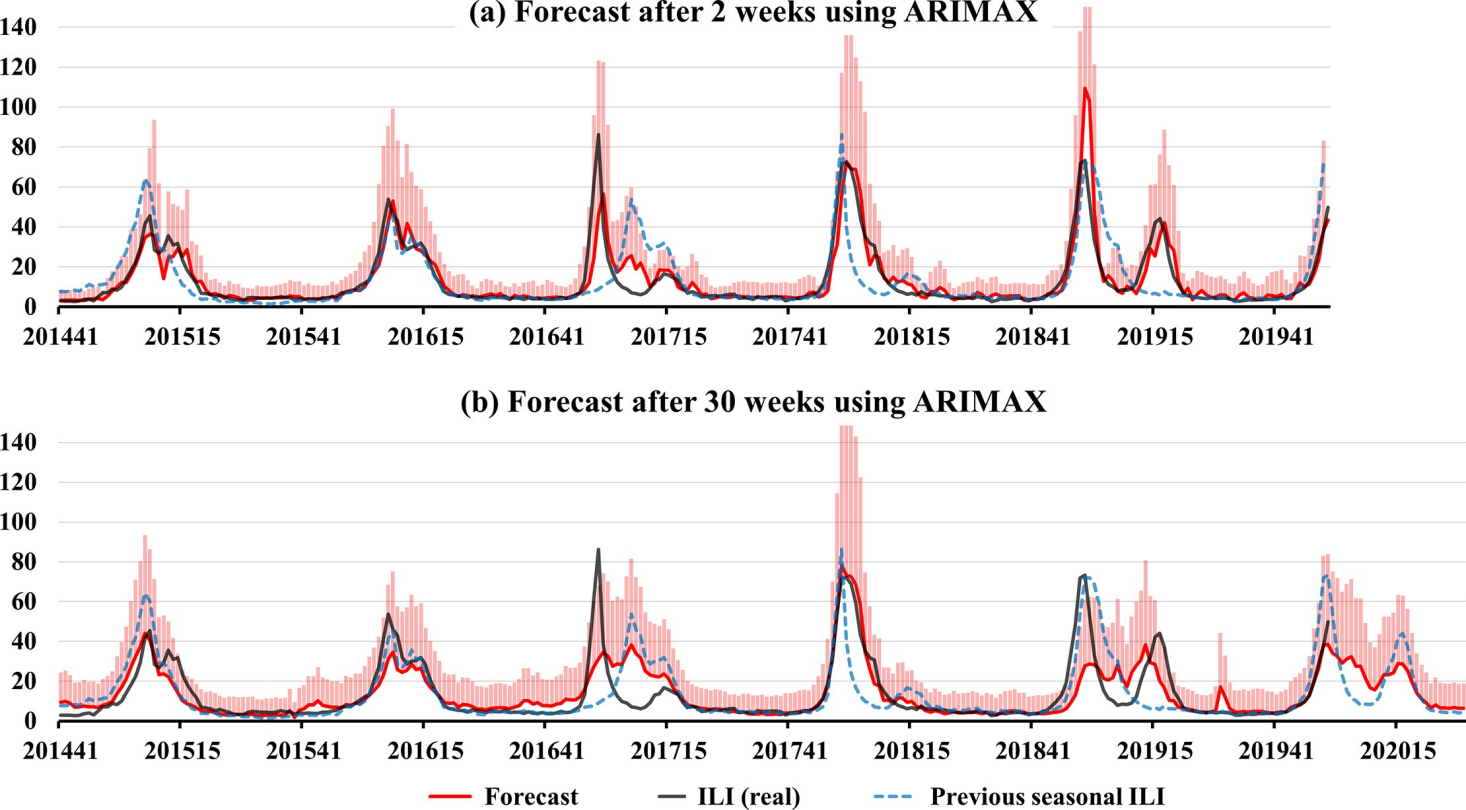
The prediction of ILI after 2 and 30 weeks in South Korea from the 41st week of 2015 to the 52nd week of 2019. The black line denotes ILI in South Korea, the red line denotes the prediction values, the dotted gray line denotes previous seasonal ILI, and the pink bars represent the 50% confidence intervals.

Table 3 shows the performance of the prediction models for seasonal ILI in South Korea after 2 and 30 weeks using ARMAX. The AIC values in Table 3 are the mean values of the ARMA models for the fixed rolling window analysis. The AIC values of the ARMA models for forecasting after 2 and 30 weeks with selected exogenous variables were 849.1 and 696.0, respectively, which were lower than those of the reference models. The *R*^*2*^ score of the prediction models after 2 weeks using the total GT was 0.789, higher than the reference value of 0.623. The *R*^*2*^ score of the prediction models after 30 weeks, using average vapor pressure and total INF in Argentina, was 0.621, higher than the reference value of 0.247. The RMSEs of the prediction models after 2 and 30 weeks were lower than the RMSEs of the reference. Fig 4 reports the prediction of ILI after 2 and 30 weeks in South Korea from the 41^st^ week of 2015 to the 52^nd^ week of 2019, with 50% confidence intervals, which indicate the forecast uncertainty.

**Table 3:** Performance of the ARMA forecast models after 2 and 30 weeks in South Korea from the 41^st^ week of 2014 to the 52^nd^ week of 2019.

ARMAX	Forecast week	Mean AIC	*R*^*2*^	RMSE	Input variables			
					Previous seasonal ILI	Total GT	Average vapor pressure	Total INF in Argentina
Reference	2 weeks	917.5	0.623	9.4	O			
	30 weeks	743.7	0.247	13.3	O			
Forecast	2 weeks	849.1	0.789	7.1	O	O		
	30 weeks	696.0	0.621	9.5	O		O	O

AIC, Akaike Information Criterion; *R*^*2*^, Coefficient of determination; RMSE, Root-mean-square error; ARMAX, Auto Regressive Moving Average including exogenous variables; ILI, Influenza-like illness; GT, Google Trends; INF, Influenza

**Fig 5:**
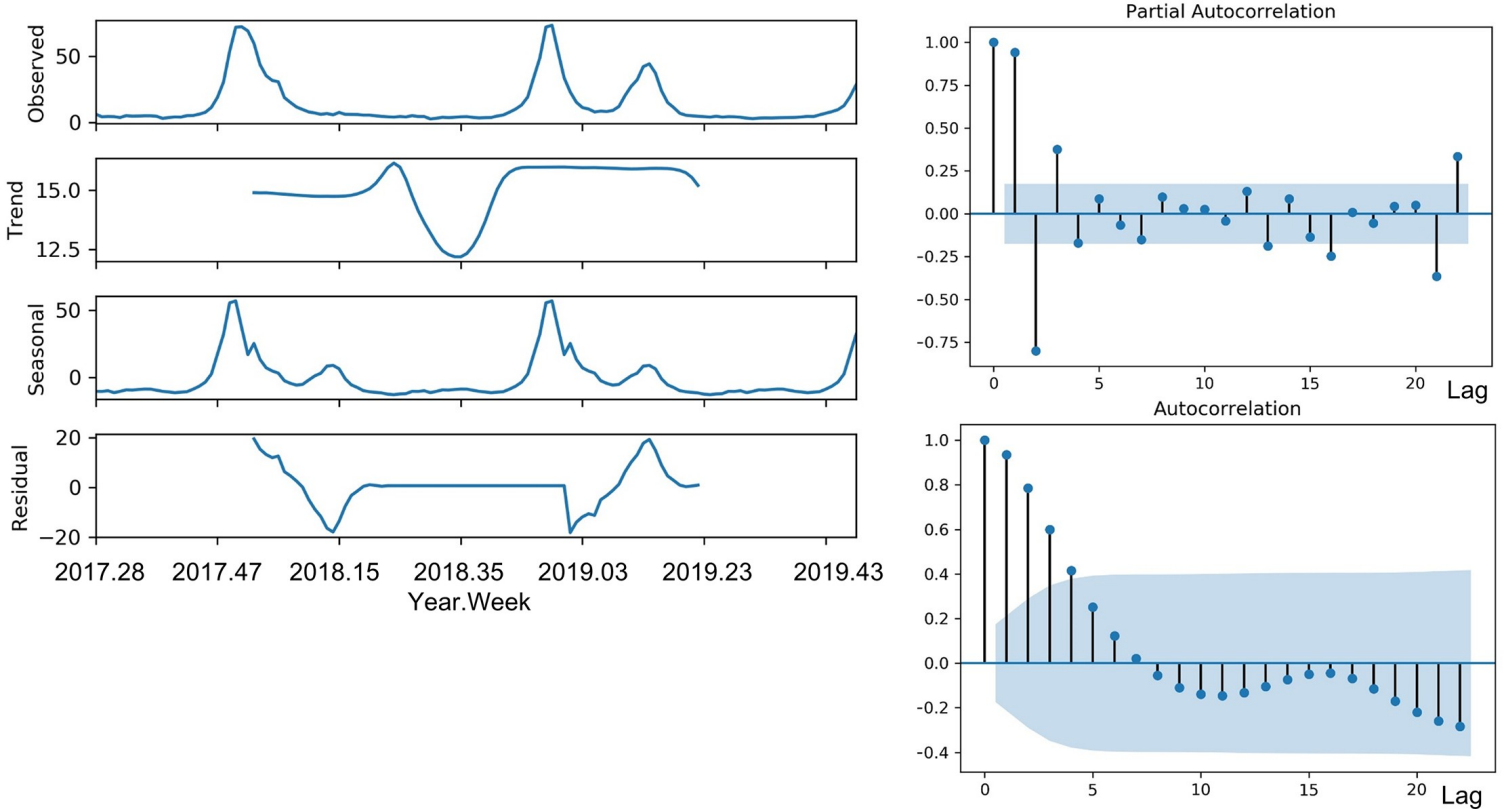
The seasonal components and coefficients of correlation of the training set for the final model.

**Fig 6:**
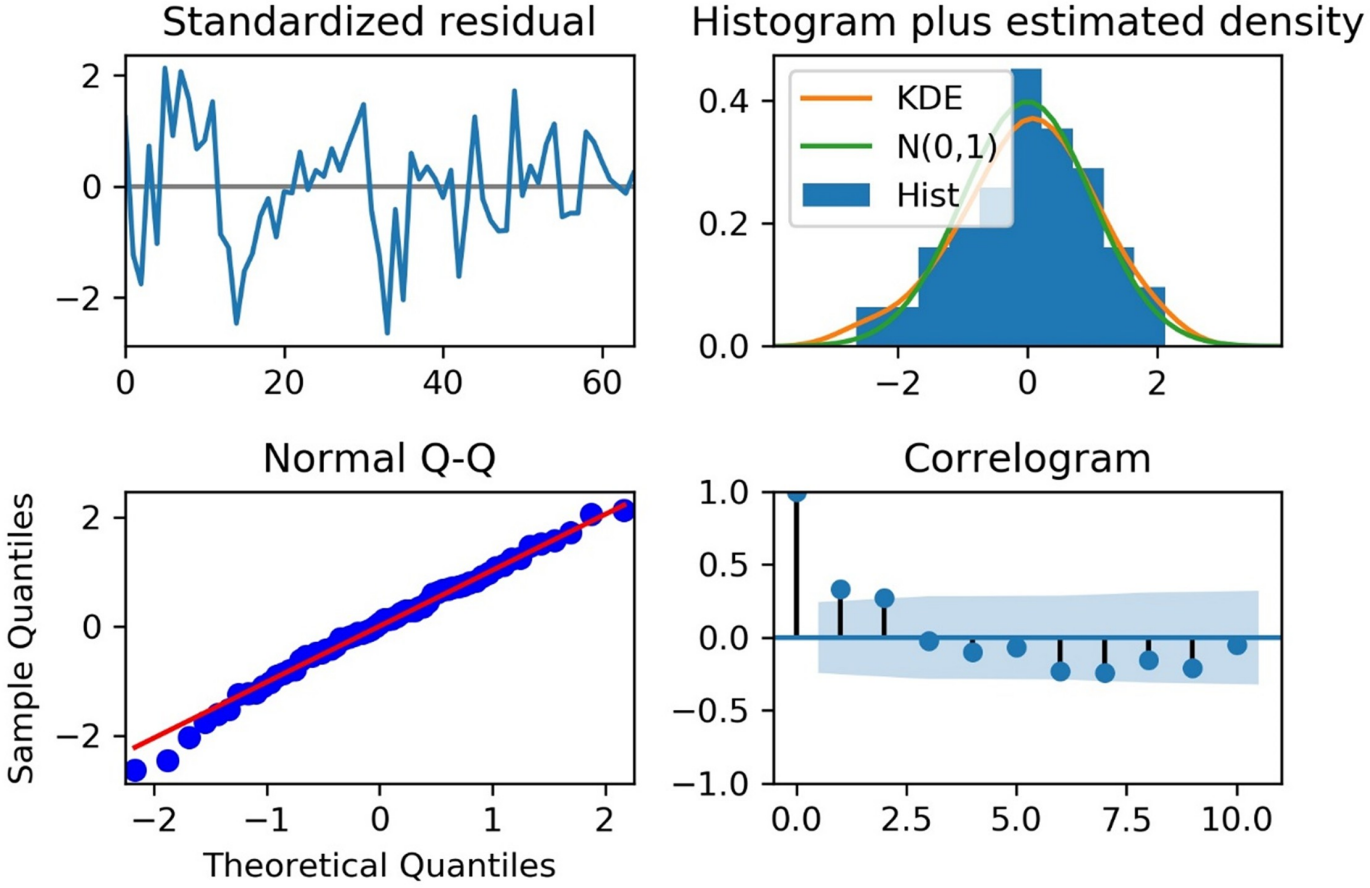
Analysis of the residuals of the final model.

**Fig 7:**
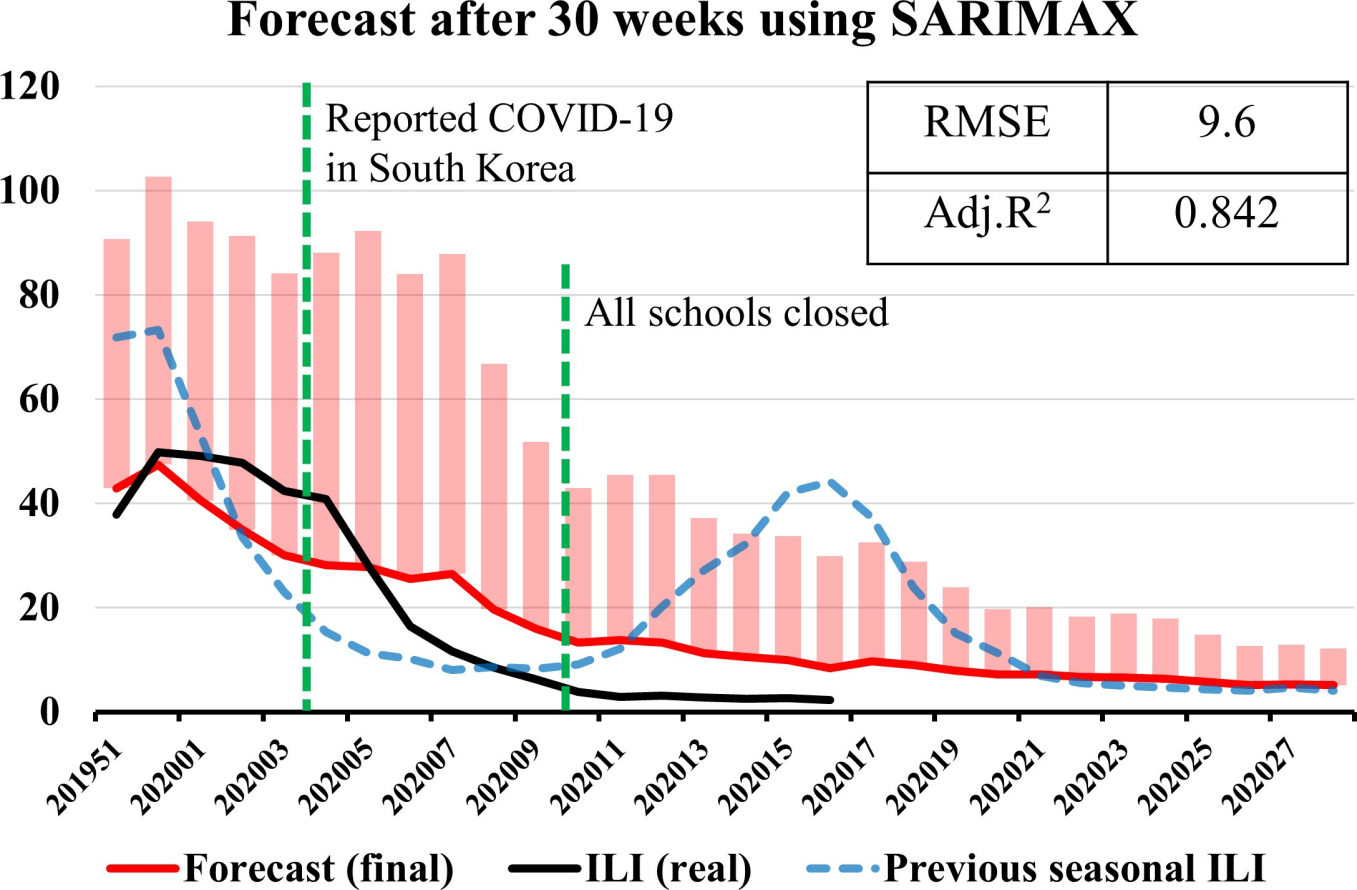
The observed real values of ILI and the forecast of the final model. The black line denotes ILI in South Korea, the red line denotes the prediction values, the dotted blue line denotes previous seasonal ILI, and the pink bars represent the 50% confidence intervals.

To construct the final model for forecasting ILI after 2 and 30 weeks at the 50^th^ week of 2019, the training set was the ILI data from the 50^th^ week of 2016 to the 50^th^ week of 2019. Fig 5 shows the seasonal components and coefficients of correlation of the training set for the final model, which are the trend, seasonal, resid attributes, ACF, and PACF. The results of the Dickey-Fuller test of the training set were -3.6 for test statistic and 0.006 for *P*-value; so, the ILI data was stationary time series, and we did not use the Box-Cox transformation for normalization. The test statistic value and *P*-value of the seasonal Mann–Kendall test were -0.871 and 0.383; there was no trend in the ILI data during the last three years. The automatically selected parameter (*p*, *q*) by AIC was 4 of *p* and 1 of *q*. Among ARMAX (*p*, *q*), ARIMAX (*p*, *d*, *q*), and SARIMAX (*p*, *d*, *q*) (*p*, *d*, *q*)_seasonal_, we selected a final model with the smallest AIC value, which was SARIMAX (4,1,1) (1,0,0)_52_ model in Table 4. Fig 6 shows the standardized residual, histogram plus estimated density, normal Q–Q, and ACF of the residual of the final model. Fig 7 shows the observed real values of ILI and the forecast of the final model from the 51^st^ week of 2019 to the 28^th^ week of 2020. The RMSE and *R*^*2*^ scores of the final model were 9.6 and 0.842, respectively. The ILI of the 2019–2020 season did not show a second wave.

**Table 4:** Evaluation criteria results for the final models after 2 and 30 weeks in South Korea using the training set for three years.

Models		AIC	Ljung-Box (*P*-value)	Jarque-Bera (*P*-value)
2 weeks	ARMAX (1,0)	911.4	45.3 (<0.01)	460.3 (<0.01)
	ARMAX (4,1)	837.2	10.9 (0.95)	1831.7 (<0.01)
	ARIMAX (4,1,1)	642.3	70.0 (<0.01)	35.1 (<0.01)
	SARIMAX (4,1,1) (1,0,0)_52_*	377.7	98.8 (<0.01)	1.9 (0.38)
	SARIMAX (4,2,1) (1,0,0)_52_	378.9	69.1 (<0.01)	0.6 (0.74)
30 weeks	ARMAX (1,0)	738.8	98.8 (<0.01)	164.4 (<0.01)
	ARMAX (4,1)	668.2	41.4 (0.80)	123.3 (<0.01)
	ARIMAX (4,1,1)	492.4	84.0 (<0.01)	17.7 (<0.01)
	SARIMAX (4,1,1) (1,0,0)_52_*	291.5	73.4 (<0.01)	13.6 (<0.01)
	SARIMAX (4,2,1) (1,0,0)_52_	305.6	104.8 (<0.01)	12.4 (<0.01)

AIC, Akaike Information Criterion; ARMAX, Auto Regressive Moving Average including exogenous variables; ARIMAX, Auto-Regressive Integrated Moving Average including exogenous variables; SARIMAX, Seasonal ARIMAX.

*We selected a final model with the smallest AIC value.

## Discussion

The current study aimed to identify variables for forecasting seasonal and short-term targets for ILI in South Korea and suggest prediction models for forecasting after 2 and 30 weeks using the variables. Among the input variables from various domains, the total GT showed the highest correlation coefficient with a week ahead. Moreover, the high correlation of seasonal influenza outbreak patterns in Argentina with those of South Korea when considering the 30-week forecast indicated that Argentina’s seasonal influenza patterns 30 weeks prior were highly correlated with the current seasonal ILI patterns in South Korea. These variables were more useful for forecasting seasonal and short-term targets than previous seasonal ILI in South Korea. The *R*^*2*^ values reported in the prediction models after 2 and 30 weeks using ARMAX indicated that the reference models could be improved using only the previous seasonal ILI.

In this study, total INF in Argentina showed a high correlation with ILI after 30 weeks in South Korea. In our previous study, total INF in Australia and Chile showed a high correlation with ILI after 22 and 28 weeks, respectively, in the United States [7]. Countries in the southern hemisphere showed different influenza patterns, and we identified those with seasonal patterns and influenza outbreaks similar to but preceding those of the reference country [7]. In Table 1, the absolute values of the time lag for Argentina and Chile in the Southern Hemisphere are higher than those for Ireland and China in the Northern Hemisphere. Therefore, the time lag for total INF in Argentina using the cross-correlation analysis was related to the latitude difference between Argentina and South Korea. Moreover, the correlation coefficient of the cross-correlation analysis for Argentina (0.717) in Table 1 was higher than those for China (0.699) and Japan (0.517), which are neighboring countries of South Korea. Although this study did not prove the causality of the correlations between countries for seasonal influenza, the seasonal influenza patterns between Argentina and South Korea were similar, indicating that influenza surveillance in Argentina can be used to predict seasonal ILI patterns after 30 weeks in South Korea.

The total GT had the highest correlation coefficient for ILI in South Korea and proved to be a powerful variable for estimating peak timing for the short-term targets, but not peak amplitude. For example, Google Flu Trends overestimated the prevalence of influenza in the 2011–2012 and 2012–2013 seasons by more than 50% in the United States [30]. During the peak flu season in 2014–2015, Google Flu Trends reported that 11% of the United States had influenza, nearly double the actual 6% reported by the CDC [30]. When flu-related events occur and are reported in the news or media, people without flu symptoms may search for flu-related keywords, which can lead to bias due to the increased volume of searches [31]. However, among variables that can be obtained in real-time, it is difficult to find a variable with a high correlation with seasonal flu patterns. Therefore, it is necessary to supplement the GT data with keyword combinations and machine learning.

The variables for air pollution were not selected to forecast ILI in South Korea. Liu et al. demonstrated that SO_2_ was positively associated with laboratory-confirmed influenza, which indicated that the number of confirmed influenza cases increased when the air concentration of SO_2_ was high [32]. The average vapor pressure has a higher correlation coefficient with ILI than those of temperature and relative humidity in South Korea. Bai et al. reported that vapor pressure was significantly associated with ILI in China; however, its correlation coefficient was lower than the correlation coefficient for temperature [33].

The real ILI values after the 5^th^ week of 2020 were lower than the forecast result of the final model in Fig 7. When patients with Coronavirus Disease-19 (COVID-19) were reported in South Korea, the Korean government recommended wearing a mask, maintaining social distance, and closing schools. These policies could have lowered influenza outbreaks as well as COVID-19.

The current study had several limitations. There were insufficient data on other potential covariates, such as the standard of the medical facilities, economic levels in South Korea and Argentina, and medical records related to the influenza virus. Further research to explain the underlying mechanisms of the relationship of influenza activity between these countries is warranted.

## Conclusions

This study identified input variables suitable for short-term and long-term predictions of ILI in South Korea by cross-correlation analysis. Although total GT had a negative time lag, it is eligible as an input variable for the two-week forecast, since the KCDC releases the ILI value of the week before the present time. Short-term predictions performed better than long-term predictions but were not suitable for predicting peak timing and amplitude. Therefore, we suggest the prediction model be used after 30 weeks in South Korea, using influenza surveillance and average vapor pressure data from Argentina. Improved predictions for seasonal ILI after 2 and 30 weeks could help the KCDC determine vaccine strategies for the next season of ILI.
